# Sustained response to onabotulinumtoxin A in patients with chronic migraine: real-life data

**DOI:** 10.1186/s10194-020-01113-6

**Published:** 2020-04-25

**Authors:** Raffaele Ornello, Simona Guerzoni, Carlo Baraldi, Luana Evangelista, Ilaria Frattale, Carmine Marini, Cindy Tiseo, Francesca Pistoia, Simona Sacco

**Affiliations:** 1 Neuroscience Section, Department of Applied Clinical Sciences and Biotechnology, University of L’Aquila, L’Aquila, Italy; 2 Medical Toxicology Unit-Headache and Drug Abuse Research Center, Department of Biomedical, Metabolical and Neural Sciences, University of Modena and Reggio Emilia, Modena, Italy; 3 Department of Clinical Medicine, Public Health, Life and Environmental Sciences, University of L’Aquila, L’Aquila, Italy

**Keywords:** Headache, Migraine, Botulinum toxin, Response, Prevention, Open-label study

## Abstract

**Background:**

Treatment with onabotulinumtoxin A (BT-A) is safe and effective for chronic migraine (CM). Several studies assessed possible predictors of response to treatment with BT-A, but there is little knowledge on the frequency and predictors of sustained response. The aim of this study was to evaluate sustained response to BT-A in patients with CM.

**Main body:**

In this prospective open-label study, 115 patients with CM and treated with BT-A were consecutively enrolled in two Italian headache centers and followed up for 15 months. Anytime responders were defined as those patients who achieved a ≥ 50% reduction in headache days during any three-month treatment cycle compared with the 3 months prior to initiation of BT-A treatment. Sustained responders were defined as those who achieved a ≥ 50% reduction in headache days within the third treatment cycle and maintained response until the end of follow-up. Non-responders were defined as those patients who never achieved a ≥ 50% reduction in headache days during the follow-up. Headache characteristics prior to BT-A treatment were assessed in order to evaluate their ability in predicting treatment response.

The 115 enrolled patients (84.3% female; median age 50 years) had a median migraine duration of 30 years (interquartile range 22–38). At the end of follow-up, 66 patients (57.4%) were classified as anytime responders. Among the 51 patients who achieved a clinical response within the third month of treatment, 33 (64.7%) were sustained responders. Patients with sustained response had a lower CM duration (median 31 vs 65 months; *P* = 0.030) and a lower number of headache days (median 25 vs 30; *P* = 0.013) at baseline compared with non-responders.

**Conclusions:**

About two thirds of patients who gain ≥50% response to BT-A within the third cycle of treatment maintain this positive response over time. More recent onset of CM and more headache-free days at baseline are associated with sustained response. We suggest not to delay preventive treatment of CM with BT-A, in order to increase the likelihood to achieve sustained clinical response.

## Background

Headache disorders are largely prevalent in the general population and constitute an important health concern [1], not only in developed but also in developing countries [2]. Among those disorders, chronic migraine (CM) is responsible for the highest levels of disability. CM is defined as the occurrence of ≥15 monthly headache days, of which ≥8 monthly migraine days, for ≥3 months [3]. The abovementioned definition remains valid even if some authors have recently proposed to consider ≥8 monthly migraine days for ≥3 months as sufficient to diagnose CM [4]. CM has a prevalence in the general population of about 2% [5] and imposes a significant burden on the patients in terms of social exclusion, isolation, anxiety, and depression, significantly lowering their quality of life [6, 7]. Onabotulinumtoxin A (BT-A) is a safe and effective preventive treatment for CM, as shown in the Phase 3 Research Evaluating Migraine Prophylaxis Therapy (PREEMPT) trials [8–12]. Several real-life studies confirmed its safety and efficacy in clinical practice [13–22] and showed that a shorter disease duration, some characteristics of headache (ocular, imploding), allodynia, and the absence of medication overuse and depressive symptoms are predictors of clinical response [23–33]. However, it is common experience to observe fluctuations in the clinical response to BT-A as to other preventive treatments. Assessing sustained response to BT-A is important to reliably quantify the benefit of the treatment on the patients’ disability and quality of life. It is especially important nowadays, because alternative treatments are available for CM, namely monoclonal antibodies acting on the calcitonin gene-related peptide (CGRP) or its receptor [34, 35].

The present study aimed to assess proportion of patients who achieve sustained response to BT-A and to establish baseline headache characteristics which predict sustained response.

## Methods

### Study population

In this multicenter, prospective, open-label study, patients were consecutively enrolled from January 2015 to July 2018 in the tertiary headache centers of Modena and L’Aquila.

The following enrollment criteria were strictly followed: 1) a diagnosis of CM according to the International Classification of Headache Disorders, 2nd [36] and 3rd [37] edition; 2) clinical eligibility to BT-A treatment, i.e. prior failure of at least two oral preventive treatments; 3) consent to be followed up for at least 15 months as per duration of the PREEMPT protocol [8, 9, 12].

Patients with medication overuse or concurrent oral preventive treatments were allowed to participate in the study, as well as patients who changed their oral preventive medications during treatment with BT-A.

### Study procedures

At the baseline visit, we recorded the patients’ demographic characteristics, including age, sex, and body mass index (BMI), by using a questionnaire. A daily headache diary was dispensed to patients to collect number of monthly headache days and days of acute medication use during the 3 months before the beginning of treatment with BT-A and throughout the follow-up. In addition, we collected the Migraine Disability Assessment Scale (MIDAS), the Headache Impact Test, 6th edition (HIT-6), and the Numerical Rating Scale (NRS) scores at baseline and every 3 months. Disability according to MIDAS score was categorized as ‘no/ little’ (score 0–5), ‘mild’ (score 6.10), and moderate/ severe’ (score ≥ 11); headache impact according to HIT-6 score was categorized as ‘no/ little’ (score 36–49), ‘moderate’ (score 50–54) and ‘substantial-severe’ (scores 55–78).

Each patient started BT-A treatment with a 155-unit ‘fixed-dose, fixed-site’ protocol, with the possibility of increasing the dose up to 195 units according to a ‘follow-the-pain’ strategy [8, 9]. BT-A treatment cycles were planned to be repeated every 3 months. The study duration was of 15 months, equivalent to 5 treatment cycles plus 3 additional months of follow-up to evaluate the effect of the 5th cycle. Each patient was encouraged to continue BT-A for at least 5 treatment cycles unless there was a treatment failure or any serious adverse event. Treatment failure was assessed after 3 treatment cycles (9 months) and was defined as a < 30% of reduction in headache days from baseline [13, 38]. All patients, even those who stopped treatment, were asked to fill in their diaries and questionnaires for the 15-month duration of the study follow-up.

### Definition of responders

‘Anytime responders’ were defined as those patients who achieved a ≥ 50% reduction in headache days during any three-month treatment cycle compared with the 3 months prior to initiation of BT-A treatment; this definition is in line with commonly accepted criteria [13, 38, 39].

‘Sustained responders’ were defined as those patients achieving a ≥ 50% reduction in headache days within the third treatment cycle and maintaining the response until the end of follow-up.

‘Non-responders’ were defined as patients who never achieved a ≥ 50% reduction in headache days during the follow-up.

Patients discontinuing BT-A treatment because of adverse events, ineffectiveness, or lack of adherence were considered as non-responders. For patients discontinuing treatment because of substantial clinical benefit, to address response status we considered the headache diaries and classified patients as above reported.

### Ethical aspects

The study was approved by the local ethics committees of both participating centers (protocol number 334/2015 for Modena and 0203392/16 for L’Aquila). Each patient signed an informed consent before enrolment.

### Statistical analysis

Categorical variables were reported as numbers and proportions while continuous variables were reported as mean ± standard deviation or median with interquartile range (IQR) as appropriate. Data from all patients, including those who discontinued treatment, were included in the analyses. Demographic and headache characteristics were compared between responders and non-responders using the chi-squared test for categorical variables and the Mann-Whitney U test for continuous variables. We did not pre-specify a sample size as it was based on available data. Statistical calculations were made using SPSS software, version 20. Statistical significance was set at *P* < 0.05.

## Results

**Fig. 1:**
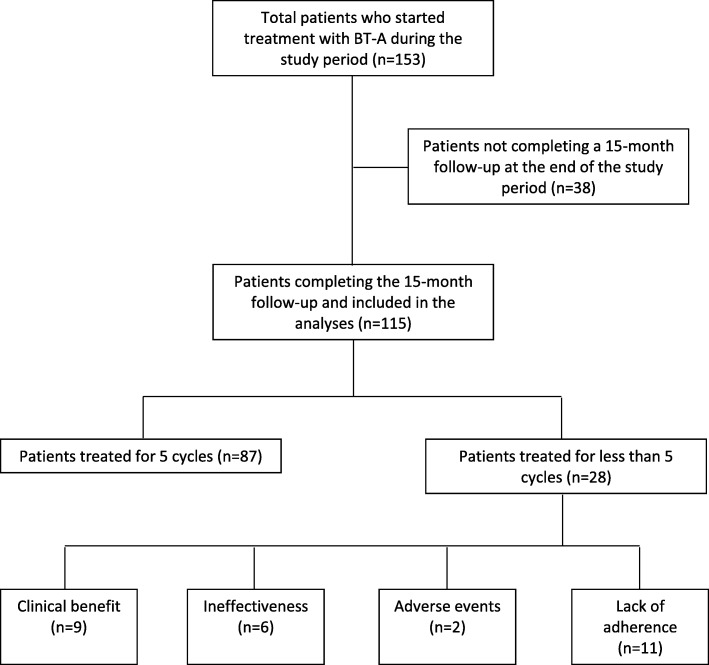
Flowchart of patient inclusion

During the study period, 115 consecutive patients were treated with BT-A and provided consent to be followed up for 15 months. Among them, 87 (75.7%) completed 5 BT-A treatment cycles; 9 (7.8%) refused to continue BT-A after the third or fourth treatment cycle for clinical benefit (all had > 75% reduction in monthly headache days); 19 additional patients stopped BT-A because of treatment failure (6; 5.2%), adverse events (2 with allergic reaction; 1.7%), or lack of adherence (11; 9.6%), (Fig. 1). All patients discontinuing treatment filled out diaries and questionnaires until the end of the study; no other patient was lost to follow-up. Patients who stopped BT-A for clinical benefit did not restart BT-A by the end of the study, nor started any other preventive treatment.

The baseline characteristics of the included patients are reported in Table 1. Most patients (84.3%) were women, while the median age was 50 years (interquartile range [IQR] 44.5–54). Eighty-nine (77.4%) patients had medication overuse and 61 (53.0%) were on concurrent preventive treatments. The median migraine duration was 30 years, while the median CM duration was 62.5 months.

**Table 1:** Baseline characteristics of the study patients (*N* = 115)

Female, n (%)	97 (84.3)
Age, median (IQR)	50 (44.5–54)
Medical history, n (%)	
Smoking	23 (20.0)
Alcohol abuse	10 (8.7)
Family history of headaches	78 (67.8)
Arterial hypertension	29 (25.2)
BMI, median (IQR)	23 (20–25)
Headache characteristics, n (%)	
Aura	19 (16.5)
Allodynia	38 (33.0)
Pain characteristics, n (%)	
Unilateral	48 (41.7)
Throbbing	74 (64.3)
Diffuse	24 (20.9)
Frontal	60 (52.2)
Temporal	59 (51.3)
Ocular	34 (29.6)
Occipital	15 (13.0)
Parietal	15 (13.0)
Vertex	9 (7.8)
Medication overuse, n (%)	89 (77.4)
Preventive treatments in history, n (%)	
Antidepressants	82 (71.3)
Anticonvulsants	69 (60.0)
Calcium antagonists	42 (36.5)
Other	8 (7.0)
Concurrent oral preventive treatments, n (%)	61 (53.0)
Migraine duration (years), median (IQR)	30 (22–39.5)
Chronic migraine duration (months), median (IQR)	62.5 (24–144)

BMI indicates body mass index; *IQR* interquartile range

Headache days and medication days both decreased from a median of 30 (IQR 25–30) at baseline to 15 (IQR 7–25; *P* < 0.001) (Table 2). The median headache intensity significantly decreased from 8 (IQR 7–9) to 5 (IQR 4–7; *P* < 0.001). The patients’ quality of life also improved, as suggested by the MIDAS score decrease from 87.5 (IQR 42.5–123.5) to 12 (IQR 3.5–51.5; *P* < 0.001) and by the HIT-6 score decrease from 65 (IQR 60–69) to 62 (IQR 56–65; *P* < 0.001).

**Table 2:** Change in study outcomes at 15-month follow-up in the 115 included patients

Outcome	Pre-treatment median (IQR)	Post-treatment median (IQR)	*P* value
Headache days	30 (25–30)	15 (7–25)	< 0.001
Medication days	30 (25–30)	15 (7–25)	< 0.001
MIDAS score	87.5 (42.5–123.5)	12 (3.5–51.5)	0.001
HIT-6 score	65 (60–69)	62 (56–65)	< 0.001
NRS score	8 (7–9)	5 (4–7)	< 0.001

HIT-6 indicates Headache Impact Test, 6th edition; *IQR* interquartile range, *MIDAS* Migraine Impact and Disability Assessment Scale, *NRS* Numerical Rating Scale

**Fig. 2:**
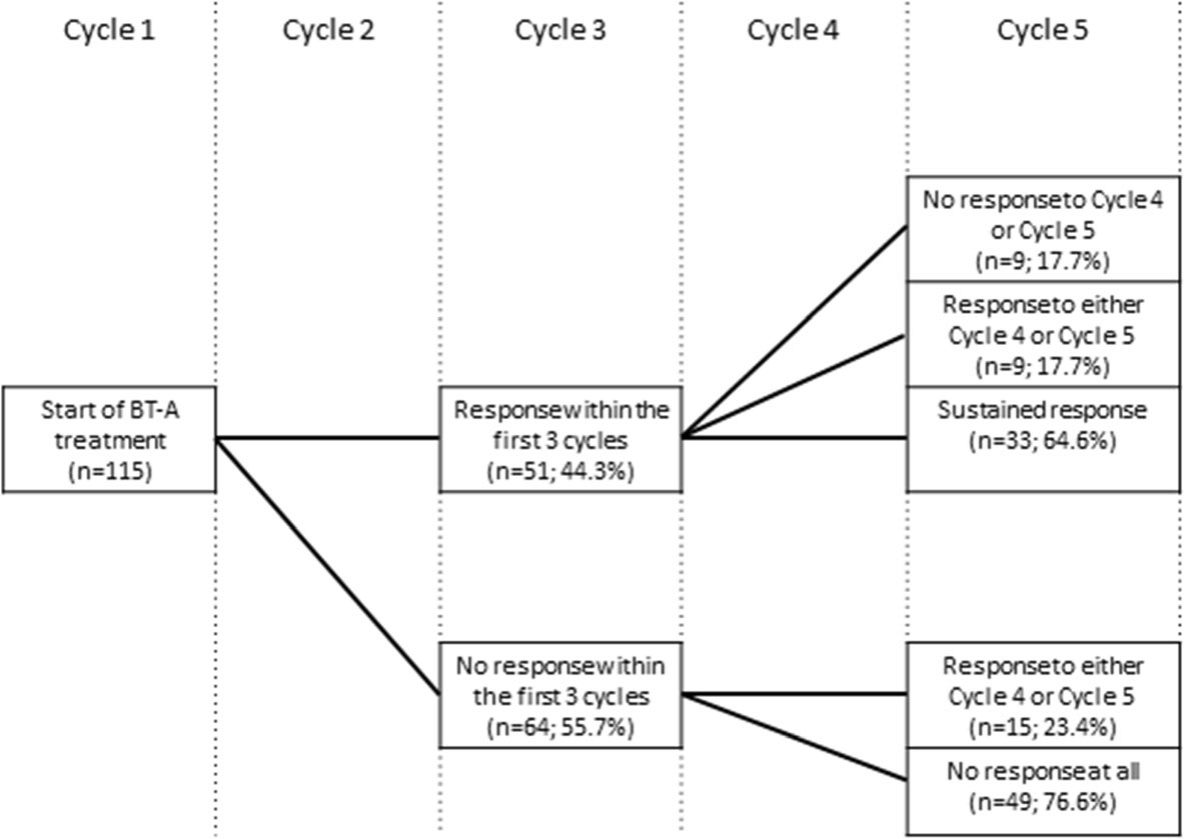
Proportion and course of response to treatment with onabotulinumtoxin A

**Fig. 3:**
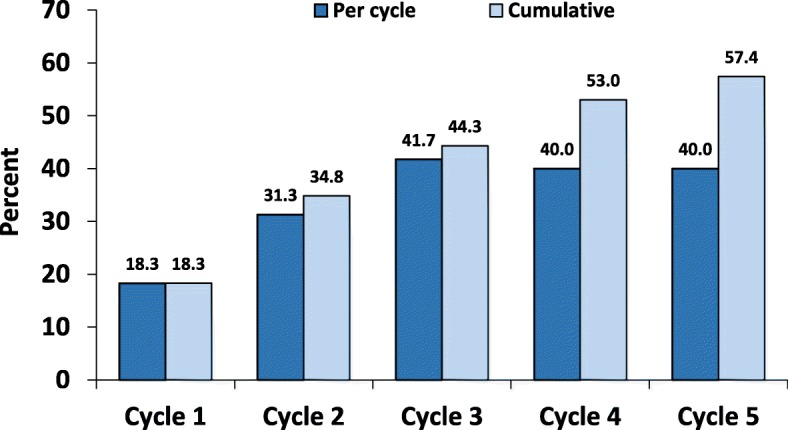
Proportion of patients with ≥50% reduction in headache days during each three-month treatment cycle compared with the 3 months prior to initiation of treatment with onabotulinumtoxin A

The proportion of responders per cycle of treatment ranged from 18.3% to 41.7% over the study period (Fig. 2). At the end of follow-up, 66 patients (57.4%) patients were classified as anytime responders and the remaining 49 patients (42.6%) were classified as non-responders. Fifty-one (44.3%) patients achieved response to treatment within the third cycle; 33 (64.7%) of them were classified as sustained responders while 18 (35.3%) had fluctuations in treatment responses over the following cycles (Fig. 3).

**Fig. 4:**
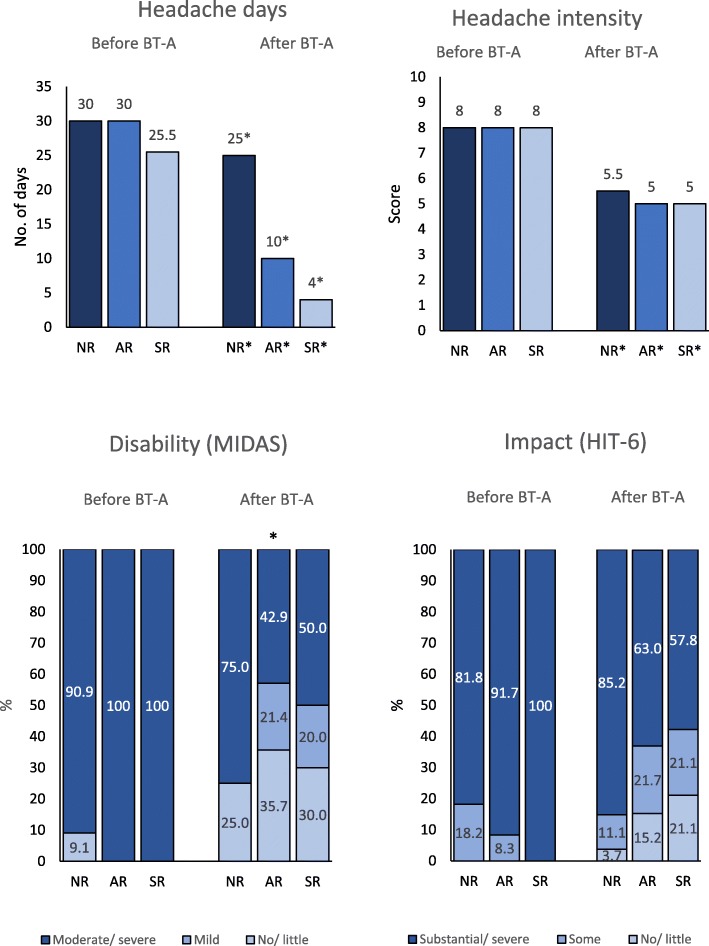
Median headache days and intensity and patients distribution according to MIDAS and HIT-6 scores during the 3 months before onabotulinumtoxin A initiation and during the final 3 months of follow-up. Results are stratified by response status. The asterisk (*) identifies significant changes compared with pre-treatment. NR indicates non-responders; AR, anytime responders; SR, sustained responders

Figure 4 shows the clinical data stratified according to response status. Not only responders, but also non-responders had a significant decrease in the number of headache days and in headache intensity as compared to baseline. The disability (MIDAS) and impact (HIT-6) of headache improved numerically but not significantly after treatment in anytime and sustained responders (Fig. 4).

Sustained responders had a lower CM duration (31 vs 49 months; *P* = 0.030) and a lower median number of monthly headache days at baseline (25 vs 30; *P* = 0.013) compared with non-responders (Table 3) while the baseline headache characteristics and changes in concurrent oral preventive treatments were similar in the two groups.

**Table 3:** Univariate comparison of the characteristics of responders vs non-responders to treatment with onabotulinumtoxin-A

	Non-responders (*n* = 49)	Anytime responders (*n* = 66)	*P* value	Sustained responders (*n* = 33)	*P* value
Age, median (IQR)	50 (42–55)	49 (44–54)	0.627	49 (45–53)	0.491
Female sex, n (%)	43 (87.8)	54 (81.8)	0.386	28 (84.8)	0.705
Migraine years, median (IQR)	31 21.5–40)	30 (22–40)	0.913	25 (20–34)	0.155
Chronic migraine duration (months), median (IQR)	65 (25–120)	49 (21–126)	0.732	31 (15.5–60)	**0.030**
Monthly headache days, median (IQR)	30 (30–30)	30 (24–30)	0.176	25 (21.5–30)	**0.013**
Medication overuse, n (%)	37 (82.2)	52 (78.8)	0.656	22 (66.7)	0.114
Aura, n (%)	11 (23.4)	8 (12.1)	0.114	3 (9.1)	0.137^a^
Allodynia, n (%)	18 (45.0)	20 (33.9)	0.265	13 (41.9)	0.796
Unilateral headache, n (%)	18 (38.3)	30 (45.5)	0.449	15 (45.5)	0.522
Throbbing headache, n (%)	30 (63.8)	44 (66.7)	0.755	21 (63.6)	0.986
Diffuse headache, n (%)	11 (23.4)	13 (20.0)	0.665	5 (15.2)	0.409^a^
Frontal headache, n (%)	23 (48.9)	37 (56.1)	0.454	22 (66.7)	0.116
Temporal headache, n (%)	26 (55.3)	33 (50.0)	0.577	17 (51.5)	0.737
Orbital headache, n (%)	13 (27.7)	21 (31.8)	0.635	7 (21.2)	0.612
Occipital headache, n (%)	7 (14.9)	8 (12.1)	0.669	4 (12.1)	0.999^a^
Parietal headache, n (%)	9 (19.1)	6 (9.1)	0.120	3 (9.1)	0.341^a^
Vertex headache, n (%)	6 (12.8)	3 (4.5)	0.112^a^	2 (6.1)	0.459^a^
Concurrent oral preventive treatments, n (%)					
At baseline	28 (57.1)	33 (50.0)	0.448	17 (51.5)	0.616
Withdrawn during treatment	4 (8.2)	4 (6.1)	0.722^a^	4 (12.1)	0.708^a^
Changed during treatment	13 (26.5)	13 (19.7)	0.386	7 (21.2)	0.582
Initiated during treatment	2 (4.1)	9 (13.6)	0.113^a^	5 (15.2)	0.111^a^

^a^Fisher’s exact test

At the end of follow-up, 40 of the 115 included patients (34.8%) had mild adverse events; the most common were local tension (*n* = 14; 12.2%), local pain (*n* = 12; 10.4%), and muscle weakness or atrophy (*n* = 11; 9.6%).

## Discussion

Migraine frequency and intensity, as well as response to treatments, undergo fluctuations as a response to internal and external triggering factors; the fluctuation between satisfactory and unsatisfactory responses to treatment might be frustrating for patients with CM. A way to improve the management of patients with migraine is to evaluate not only the response to treatments at a given time point, but also sustained benefit over time. To our knowledge, our study is the first to address the concept of ‘sustained response’ to BT-A.

We found that more than half of patients had a ≥ 50% reduction in headache days from baseline after at least one cycle of BT-A treatment and that about two thirds of patients who achieved a ≥ 50% reduction in headache days within the third treatment cycle had a sustained response to BT-A. Our results should be read in the light of a difficult-to-treat study population of two tertiary level headache centers, who had a high mean age, a long history of migraine and of CM, and a high prevalence of medication overuse. The proportion of patients with sustained response to BT-A treatment would likely be higher in less difficult-to-treat populations.

In our real-life setting, patients who had higher likelihood of sustained response were those with a shorter CM duration and lower burden of headache days. No other studies have evaluated predictors of sustained response and we can compare our findings only with those studies who have evaluated anytime response [23–33] (Table 4). Anyhow, our data suggest that some factors which predicted short-term response, including short CM duration, are predictors of sustained response [30, 32] (Table 4). The comparable studies assessing the response to BT-A had heterogeneous treatment protocols, follow-up durations, and patient populations (Table 4). Three studies found that shorter migraine duration predicted a favorable response to BT-A [23, 30, 32], in line with our finding regarding CM duration. Notably, a favorable response to BT-A was not predicted by the overall duration of migraine, but rather by the duration of its chronic stage. Hence, the detection of migraine chronification is crucial to provide proper access to specialist care and the most effective options for CM treatment as early as possible. Other studies found that ocular or imploding headache predicted response to BT-A [24, 26, 27, 29], while in our study the pain location did not influence BT-A response. Medication overuse predicted unfavorable response to BT-A in one study [33], which contrasts with our findings; however, a further study supports our finding of no difference in response between patients with and without medication overuse [40]. The optimal management of CM associated with medication overuse is in fact controversial. Medication overuse is not a contraindication to BT-A treatment for CM and does not preclude its effectiveness [39, 41]. However, detoxication prior to BT-A initiation may per se determine reversion of CM and may improve the efficacy [42]. In our study population, detoxication prior to BT-A initiation was not mandatory; however, we cannot exclude that an extensive use of detoxication might increase the proportion of anytime and sustained responders.

**Table 4:** Characteristics of real-life studies of onabotulinumtoxin A for the treatment of migraine assessing predictors of treatment response

Study	Country	N	% women	Years of age, mean ± SD (range)	% CM	Years from CM onset, mean ± SD (range)	% medication overuse	BT-A dose range (U)	Max follow-up (months)	Definition of response to BT-A	% BT-A responders	Significant predictors of response to BT-A
Eross, 2005 [23]	USA	61	90.0	46.5 (15–81)	77	NR	NR	25–100	4	≥50% reduction in headache-related disability	62.0	Short duration of migraine
Jakubowski, 2006 [24]	USA	27	92.6	41.9 ± 1.7	NR	NR	NR	100	12	> 80% reduction in monthly headache days	51.9	Imploding and ocular migraine
Mathew, 2008 [25]	USA	71	90.1	(19–69)	100.0	NR	NR	100	7	≥50% reduction in headache frequency and MIDAS score	76.1	Unilateral location, allodynia, pericranial muscle tenderness
Burstein, 2009 [26]	USA	82	84.1	50.9 ± 1.2 (21–75)	67.0	NR	NR	NR	NR	> 67% reduction in monthly headache days	45.1	Imploding and ocular migraine
Kim, 2010 [27]	USA	18	94.4	50.9 (26–80)	NR	NR	NR	25–300	3	≥50% reduction in headache frequency	77.8	Imploding and ocular migraine
Bumb, 2013 [28]	Switzerland	111	85 (responders) 77 (nonresponders)	47 (responders) 52 (nonresponders)	66.0 (responders) 62.5 (nonresponders)	NR	NR	100	NR	Patients undergoing ≥3 treatments	57.7	None
Lin, 2014 [29]	Taiwan	94	84.0	47.6 ± 13.6 (20–85)	100.0	8.1 ± 8.3 (1–40)	19.1	75–155	3	≥30% reduction in headache frequency	39.4	Ocular-type headache
Lee, 2016 [30]	South Korea	70	85.7 (responders 85.7 (nonresponders)	48.0 ± 13.6 (responders) 46.9 ± 10. (nonresponders)	100.0	Median 10 (IQR 5–17) (responders Median 15 (IQR 10–25) (nonresponders)	47.6 (responders) 50.0 (nonresponders)	155	1	≥50% reduction in headache days, moderate-to-severe headache days, or acute medication intake	60.0	Shorter disease duration, higher MCA/ICA index at TCD
Dominguez, 2017 [31]	Spain	62	97.9 (responders 93.3 (nonresponders)	51.6 ± 9.1 (responders 39.4 ± 12.0 (nonresponders)	100.0	1.5 ± 1.0 (responders) 1.8 ± 1.3 (nonresponders)	NR	155	6	≥50% reduction in frequency of headache	75.8	Higher plasma PTX3 and CGRP levels
Dominguez, 2018 [32]	Spain	725	85.8	46.8 ± 12.0	100.0	1.7 ± 1.6	58.2	NR	12	≥50% reduction in monthly headache days	79.3	CM duration, unilateral headache, combined symptomatic treatment, headache-related disability, intensity of headache
Schiano di Cola, 2019 [33]	Italy	84	72.6	48.0 ± 9.7	100.0	10.1 ± 6.6	65.5	155–175	12	≥30% reduction in monthly headache days	82.6	Medication overuse, depressive symptoms

BT-A indicates botulinum toxin A; *CM* chronic migraine, *ICA* internal carotid artery, *IQR* interquartile range, *MCA* middle cerebral artery, *NR*not reported, *TCD* transcranial Doppler

The present study has several strengths. The study sample included patients with CM from two tertiary headache centers with experienced personnel. Besides, patients received a homogeneous treatment protocol whose response was assessed using standardized criteria. However, the present study is limited by the lack of assessment of migraine comorbidities, especially the psychiatric ones, which negatively affect the course of migraine [43] and might have a negative impact also on BT-A treatment [33]. Data on psychological treatments, including behavioral therapy [44], which might have benefited some patients were not collected in this study. Besides, changes in oral preventive treatments during treatment with BT-A in some patients might have limited the homogeneity of the study population; however, this limitation is common to all real-life studies, in which the efficacy of a drug is assessed on top of the best practice. Changes in concurrent oral preventive treatments did not differ according to response status in our population (Table 3); however, we cannot exclude that non-significant results were due to small numbers. As a further limitation, we could not assess whether BT-A was more effective on migraine than on headache days, as we did not distinguish between them. Prior response to acute medications and especially triptans was not explored, such as the information regarding the imploding or exploding nature of headache, thus limiting the comparability of the present study data with those of other studies [24, 26]. We chose a 15-month follow-up to align with the 5 treatment cycles of the PREEMPT protocol; however, longer follow-up periods are needed to assess the possible long-term benefits of BT-A in sustained responders. Lastly, we chose a cutoff of ≥50% reduction in headache days to identify responders; a relevant proportion of patients might have experienced a clinical benefit from BT-A treatment even if not fulfilling the criteria to be defined as a ‘responder’ in our study (Fig. 2). Previous data suggest that even a ≥ 30% reduction in headache days might be considered relevant to patients [13], while such a reduction identified patients as ‘non-responders’ according to our study criteria. Further studies are needed to find a better definition of ‘response’ to BT-A treatment, which takes into account the patients’ quality of life.

## Conclusion

According to our data, about two thirds of patients who achieve a clinically relevant response to BT-A within the third cycle of treatment maintain the response over time. More recent onset of CM and more headache-free days at baseline are associated with sustained response. Therefore, we suggest not to delay preventive treatment of CM with BT-A, in order to increase the likelihood of sustained clinical response.

## Data Availability

Anonymized data operated or analyzed during this study are available from the Authors upon reasonable request.

## Abbreviations

BMI: Body mass index
BT-A: Onabotulinumtoxin A
CGRP: Calcitonin gene-related peptide
CI: Confidence interval
CM: Chronic migraine
HIT-6: Headache Impact Test, 6th Edition
IQR: Interquartile range
MIDAS: Migraine Impact and Disability Assessment Scale
NRS: Numerical Rating Scale
PREEMPT: Phase 3 Research Evaluating Migraine Prophylaxis Therapy
